# Electrochemical detection of Cd(ii) ions in complex matrices with nanopipets†

**DOI:** 10.1039/d1ra07655h

**Published:** 2022-01-05

**Authors:** Muzammil M. N. Ahmed, Faieza S. Bodowara, Wendy Zhou, Juliana F. Penteado, Jessica L. Smeltz, Pavithra Pathirathna

**Affiliations:** Department of Biomedical & Chemical Engineering & Sciences, Florida Institute of Technology 150 W. University Blvd Melbourne FL 32901 USA ppathirathna@fit.edu

## Abstract

Heavy metal contamination and its detrimental health effects are a growing concern globally. Several metal mitigation systems and regulatory approaches have been implemented to minimize the negative impacts on human health. However, none of these function at maximum efficiency, mainly due to the lack of accurate information about metal speciation. Therefore, there is a critical need to develop novel, cheap, efficient, and robust metal detecting sensors. In this study, we describe the application of a nanopipet based electrochemical sensor to detect aqueous Cd(ii) ions. The inner radius of our nanopipets is ∼300 nm, and the fundamental mechanism behind our sensor's response is ion transfer between two immiscible electrolyte solutions (ITIES). The absence of redox behavior makes ITIES an excellent, attractive electrochemical tool to study various ions in aqueous solutions. In this study, we used 1,10-phenanthroline as our ionophore in the organic phase (dichloroethane) to facilitate the transfer of Cd(ii) ions from the polar aqueous phase to the less polar organic phase. Unlike previous studies, we characterized our nanopipet in complicated matrices, including, but not limited to, tris buffer and artificial seawater. We performed quantitative assessments to determine our sensor's limit of detection, stability, sensitivity, and selectivity. We further show that our nanosensor can detect free Cd(ii) ions in the presence of strong complexing agents such as ethylenediaminetetraacetic acid, 2,3-dimercaptosuccinic acid, *etc.* We quantified the concentration of free Cd(ii) ions in a water sample collected from a local lagoon. Thus, we showcased the power of our nanopipets to act as a robust, accurate, and efficient speciation sensor to detect Cd(ii) ions in environmental samples.

## Abstract 2

A nanopipet electrochemical sensor was successfully utilized to detect free Cd(ll) ions in complicated matrices and real environmental water samples.

## Graphical abstract

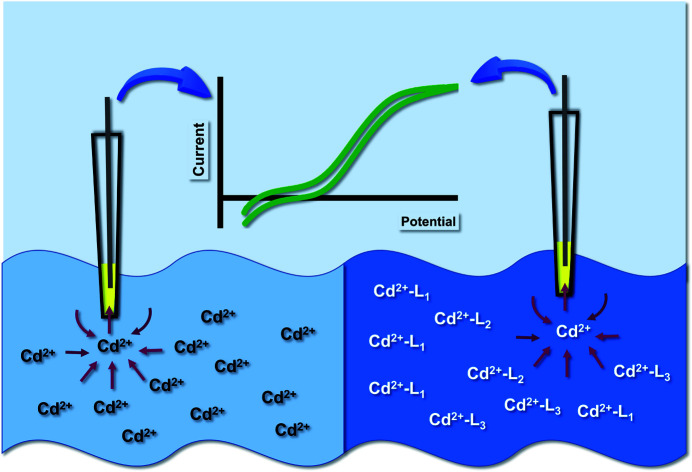

## Introduction

Exposure to heavy metals is a global health concern that results in various deleterious and harmful effects. Thus, regulation agencies such as the World Health Organization and Environmental Protection Agency^1^ have determined permissible levels of heavy metals in drinking water, food, paint, and other sources, to ensure regulation in the environment; therefore minimizing human exposure. Among different toxic metal ions, Cd(ii) is a highly poisonous heavy metal largely present in industrial effluents. Anthropogenic Cd(ii) sources include nickel–cadmium batteries, cigarettes, electroplating, and paints. Despite the rapid increase of Cd(ii) usage across the globe, there is no efficient recycling method for Cd-containing compounds; thus, humans are at a high risk of exposure to Cd(ii) *via* multiple sources. Accumulation of Cd(ii) in the human body can cause a variety of detrimental health-hazardous such as cell proliferation, apoptosis, chromosomal mutations, and damage to vital organs, including the kidneys, lungs, and liver.^2,3^

Traditionally metal analysis has been primarily performed with non-electrochemical techniques such as spectroscopy,^4,5^ chromatography,^6,7^ and colorimetry.^8^ Although these tools are compelling, they are not well-suited for on-site measurements due to the sophisticated, physically large instruments, extensive labor, and analysis time. In contrast, electrochemical techniques offer an excellent platform for real-time *in situ* metal analysis. These utilize small, robust, cheap electrodes that non-experts can easily handle, in addition to having faster response times. Chen *et al.* designed a novel electrochemical sensor by depositing BiSn nanoparticles on a glassy carbon electrode (GCE) to detect Cd(ii) using differential pulse stripping voltammetry.^9^ Because the authors increased the surface area by integrating nanoparticles onto the carbon surface, they achieved an excellent limit of detection (LOD). Abdallah and colleagues recently developed another GCE-based electrode by depositing ion-imprinted polymer.^10^ Using anodic stripping voltammetry, the authors detected Cd(ii) in biological samples. However, conventional electrochemical techniques such as cyclic voltammetry, anodic stripping voltammetry, and linear sweep voltammetry require the target analyte to easily undergo the oxidation–reduction process. Furthermore, they often need complicated fabrication processes, thus limiting the versatility of these applications.

In this respect, electrochemistry at the liquid–liquid interface has emerged in recent decades into an active branch of electroanalytical chemistry. An ionophore is typically added to the organic phase to facilitate the ion transfer across the water–organic interface in the presence of an applied external voltage.

While most researchers take advantage of ion transfer at the interface between two immiscible electrolyte solutions (ITIES) to study more complicated biological molecules such as proteins, few have reported studies with simple yet toxic metal ions.^11^ Wilke and Wang reported a thermodynamic study of Cd(ii), Cu(ii), and Pb(ii) ion transfer across a water/nitrobenzene interface using ETH1062 as the ionophore with microITIES.^12^ Benvidi *et al.* characterized Cd(ii) transfer across water/1,2-dichloroethane (DCE) using a different microITIES geometry in the presence of 1,10-phenanthroline (phen).^13^ In contrast to microITIES, Bingol and Atalay studied the transfer mechanism of Cd(ii) ions across an interfacial area of 0.27 cm^2^ in the presence of a neutral ionophore 4′-morpholinoacetophenone-4-phenyl-3-thiosemicarbazone.^14^ Lee *et al.* described a detailed study on Cu(ii) ion transfer across water/1,2-DCE with picolinamide-phenylenevinylene.^15^ These studies employed an interface of ∼20–30 μm or above between the aqueous and organic phase, revealing exciting findings. Nano-ITIES shows superior electrochemical performances over micro-ITIES leading to enhanced mass transport and low ohmic drop. More recently, Chen *et al.* introduced a new tris(crown ether) ionophore for the assisted ion transfer of metal ions at nano-ITIES.^16^ These reported studies at micro-ITIES and nano-ITIES were performed in simple matrices where the metal of interest exists primarily in the unbound state.

More precisely, metals are found in numerous bound forms with naturally present complexing agents in biological and environmental samples. In this work, we utilized a nano-ITIES based electrochemical sensor to detect free Cd(ii) ions in intricate matrices where Cd(ii) is initially bound with strong chelating agents including, but not limited to, ethylenediaminetetraacetic acid (EDTA), 2,3-dimercaptosuccinic acid (DMSA), and diethylenetriaminepentaacetic acid, (DTPA). We first characterized our nano electrochemical sensor in KCl and then in a more complex matrix resembling artificial seawater (ASW). We incorporated phen as the ionophore in our sensor as it shows the best sensitivity among all the reported ionophores for Cd(ii) detection with ITIES.^13^ Furthermore, we quantified the dissolved Cd(ii) ions in a water sample collected from the Indian River Lagoon, Melbourne, FL. To the best of our knowledge, this is the first time reporting the use of nano-ITIES based electrochemical sensors to study the speciation of Cd(ii) in a complex matrix and actual environmental samples. This study provides excellent insights into future applications of nano-ITIES electrochemical sensors, particularly in detecting heavy metals in environmental and biological samples as implantable sensors.

## Materials and methods

### Chemicals

All chemicals were purchased from Sigma-Aldrich (St. Louis, MO) unless otherwise specified. CdCl_2_ was used as the Cd(ii) source. Cd(ii) solutions were prepared in different matrices, including KCl (0.3 M), ASW, tris buffer, and phosphate buffer solution (PBS). Tris buffer was composed of tris hydrochloride (15 mM), NaCl (140 mM), KCl (3.25 mM), CaCl_2_ (1.2 mM), NaH_2_PO_4_ (1.25 mM), MgCl_2_ (1.2 mM), and Na_2_SO_4_ (2.0 mM) at pH 7.4. The composition of ASW was NaCl (402 mM), MgCl_2_ (48 mM), Na_2_SO_4_ (26 mM), and HEPES (10 mM) at pH 7.0. EDTA, DMSA, DTPA, and nitrilotriacetic acid (NTA) were used as model ligands to prepare Cd(ii)–ligand samples by mixing Cd(ii) and ligand in a 1 : 1 ratio in ASW at pH 7. CuSO_4_.5H_2_O, Fe(NO_3_)_3_·9H_2_O, FeSO_4_·7H_2_O, Ni(NO_3_)_2_·6H_2_O, Co(OOCCH_3_)_2_·4H_2_O, CaCO_3_, MgCl_2_·6H_2_O, PbCl_2_ were used as the sources for the selectivity test in KCl.

### Electrode fabrication

Borosilicate glass capillaries (O.D. = 1.0 mm, I.D. = 0.58 mm, Sutter Instruments, CA, USA) were pulled using a P-2000 laser puller (Sutter Instruments, CA, USA) to obtain nanopipets with a tip diameter of ∼600 nm. Nanopipets were imaged with a scanning electrochemical microscope, JEOL JSM-6380LV (JEOL Ltd, Tokyo, Japan), to accurately measure the pipet tip diameter (Fig. S1 in ESI†). The inner walls of the pulled pipets were silanized with chloromethylsilane prior to being filled with the organic phase. The silanization of glass pipettes is crucial^17^ in experiments where the organic phase is placed inside a glass pipette. The inner wall of the glass pipets is hydrophilic; thus, the outer aqueous phase penetrates inside the pipet and moves the organic phase upward from the tapered end, altering the electrochemical measurements. This issue is avoided by depositing a hydrophobic material such as chloromethylsilane on the inner walls of the glass pipets, hence making it more hydrophobic and resistive to aqueous solutions. Here, the pipets (maximum of 6 pipets at once) were placed inside a desiccator connected to a vacuum pump. After sufficient vacuum was established inside the desiccator, chlorotrimethylsilane (500 μL) was introduced for 30 min to 1 h. The silanization time varied depending on the relative humidity and the temperature of the surroundings. Silanized nanopipets were filled with the organic phase. The organic phase (dichloroethane) consisted of 10 mM 1,10-phenanthroline (phen) as the ionophore and tetradodecylammoniumtetrakis(pentafluorophenyl)borate (TDDATFAB, 0.1 M) as the electrolyte. TDDATFAB was synthesized as previously described.^18^

### Electrochemical experiments

All electrochemical experiments were conducted with CHI660E potentiostat (CH Instruments, TX, USA) in a three-electrode system using a lab-built Ag/AgCl electrode as the reference electrode and a Pt wire (Alfa Aesar, MA, USA) as the counter electrode. Each experiment was conducted with at least 4 nanopipets in triplicate (at least 12 runs in total).

## Results and discussion

### Cyclic voltammogram of Cd(ii) at nano-ITIES

Ion transfer across ITIES was first introduced by Koryta.^19^ The fundamental mechanism of electrochemistry at ITIES is that ion transfer is ruled by the ion's Gibbs energy of transfer for a specific aqueous-organic solvent system. Further, ions (with z+ charge) are transferred across the interface by imposing a potential difference (using a potentiostat) greater than the Gibbs energy for transfer between the two phases (eqn (1)), and the resultant current can be measured as a function of applied potential.

1 I^z+^_(aq)_ ⇌ I^z+^_(org)_

2 I^z+^_(aq)_ + L_(org)_ ⇌ [I − L]^z+^_(org)_

When the Gibbs energy for ion transfer is too high (compared to that of the background electrolyte solution), the presence of a suitable ionophore, L, in the organic phase lowers the required applied energy *via* the external circuit by forming a stable metal–ionophore complex (eqn (2)). Moreover, the second approach, facilitated ion transfer, provides greater selectivity for a particular target ion in the presence of a mixture of other ions. The steady-state current, *i*_d_, obtained for ion transfer at nano ITIES formed at the tip of a nanopipette can be equated as follows (eqn (3)),

3 *i*_d_ = 4*xzFDCr*

where *z* is the charge of the analyte, *C* is the concentration, *D* is the diffusion coefficient in the phase of origin, *r* is the radius of the pipette, *x* is a parameter that accounts for the thickness of the glass wall, and *F* is the Faraday constant.

**Fig. 1:**
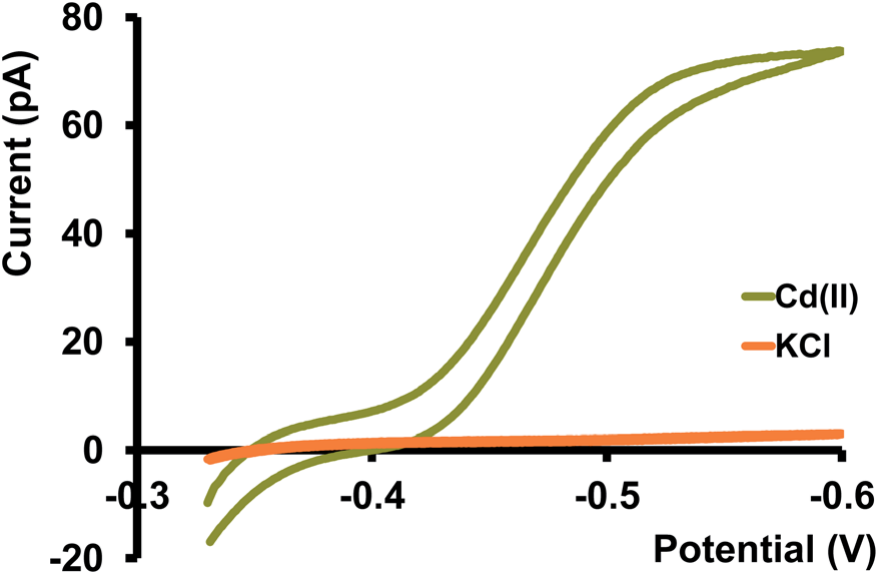
Yellowish-green trace is a representative CV of Cd(ii) transfer between water and DCE at 10 mV s^−1^. Aqueous phase: 400 μM Cd(ii) in 0.3 M KCl. Organic phase: 10 mM phen and 0.1 M TDDATFAB. Orange trace represents the background CV obtained with KCl only under the same experimental conditions.

Before moving into more complicated matrices, we tested our nanopipet ITIES sensor with Cd(ii) dissolved in a simple electrolyte solution, KCl. Upon optimizing the potential window for the detection of Cd(ii) we didn't observe any cyclic voltammograms (CV) in the absence of phen (data not shown here). We obtained a sigmoidal-shaped CV with an *E*_1/2_ value of −0.46 V after adding excess phen to the organic phase (Fig. 1). Since the preliminary work by Koryta,^19^ it was well established that the presence of ionophore in the organic phase lowers the solvation energy of the hydrophilic metal ions, subsequently reducing the Gibbs energy for the transfer across ITIES, resulting in a CV at a less negative potential. Conversely, in the absence of such an ionophore, the transfer energy of metal ions surpasses or overlaps with the energy of the ions in the background electrolyte; hence a well-resolved CV is not obtainable.

**Fig. 2:**
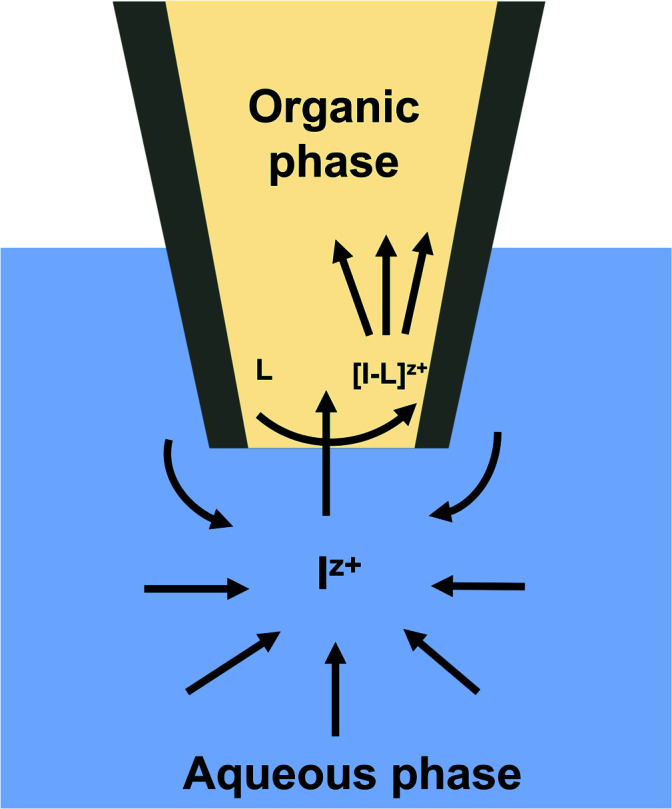
Scheme of facilitated ion transfer across two immiscible electrolyte solutions. Here, I^z+^ represents an ion with z+ charge in the aqueous solution (Cd(ii) in this study), and L represents the ionophore added into the organic phase (phen in this study) that makes a complex with I^z+^. The arrows represent the direction of the forward ion transfer of the species that contribute to the voltammetric response.

Different mass transport mechanisms at nano-ITIES define the shapes of both the forward and backward waves in a CV. In general, when excess ionophore is present in the inner organic solution and a simple ion is present in the outer aqueous solution, ingress transfer is controlled by hemispherical diffusion ( Fig. 2); thus, a steady-state sigmoidal forward wave results.^20^ Subsequently, the egress transfer of the ions or ion–ionophore complexes from the inner solution to the outer solution is controlled by linear diffusion; hence, a peak-shaped backward wave results.^13^ However, in more recent studies, steady-state sigmoidal CVs for both ingress and egress transfer at micropipet and nanopipet ITIES have been reported^16,21^ similar to our CV shown in Fig. 1. The lack of a peak on the backward wave is due to the steady-state nonlinear mass transport in the inner solution as a result of the unique tip geometry of the glass pipet. Rodgers and Amemiya used finite elemental simulations to show that the steady-state sigmoidal wave could be achieved for egress transfer with a small tip inner angle at tapered glass pipets.^20^ Establishing a mass-transport controlled steady-state voltammogram at nano-ITIES is very important, particularly in studying the kinetics of ion transfer. We found Cd(ii)'s diffusion coefficient in the aqueous phase to be 8.8 × 10^−6^ cm^2^ s^−1^, which is in close agreement with the literature reported values.^13,22^

**Fig. 3:**
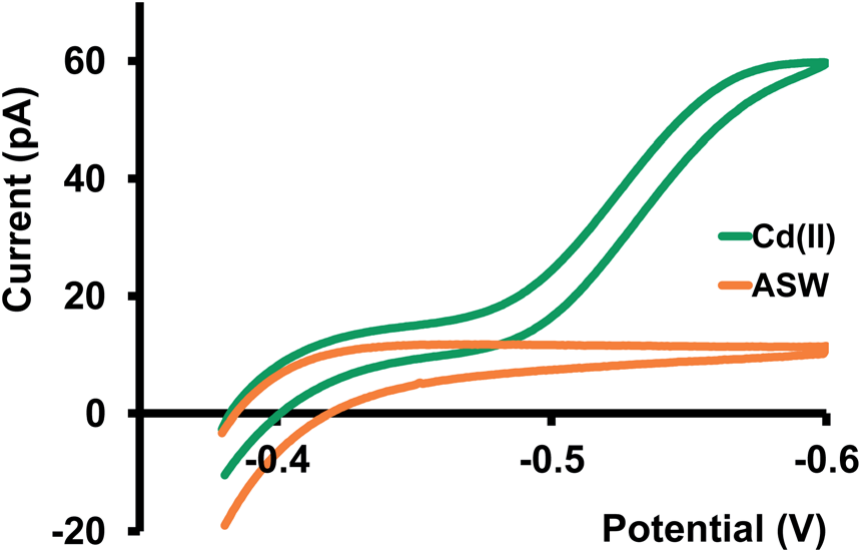
Green trace is a representative CV of Cd(ii) transfer between water and DCE at 10 mV s^−1^. Aqueous phase: 400 μM Cd(ii) in ASW buffer, organic phase: 10 mM phen and 0.1 M TDDATFAB. Orange trace represents the background CV obtained with ASW only under the same experimental conditions.

We then tested our sensors in three different buffer solutions at pH 7; tris buffer (resembles artificial cerebellum fluid), PBS (Fig. S2 and S3 in ESI†), and a buffer solution designed to mimic the composition of seawater^21^ (Fig. 3). Among all three buffer solutions, the steady-state current we obtained in ASW is most similar to that in KCl; thus, the *D* in ASW, 8.5 × 10^−6^ cm^2^ s^−1^, is more or less identical to the *D* in KCl. As predicted for the PBS solution, we obtained a low Cd(ii) transfer response. We attribute this mainly to the formation of an insoluble Cd-phosphate complex.

Furthermore, nanoscopic particles of this insoluble complex can interfere with our nano-ITIES, thus blocking the passage for Cd(ii) transfer. Response in tris buffer was not as low as in PBS; however, it wasn't as high as in KCl or ASW, resulting in a lower diffusion coefficient, 4.5 × 10^−6^ cm^2^ s^−1^. Moreover, we observed a relatively larger background current in the tris buffer, presumably due to the transfer of its background electrolyte ions.

### Calibration, stability, and selectivity

**Fig. 4:**
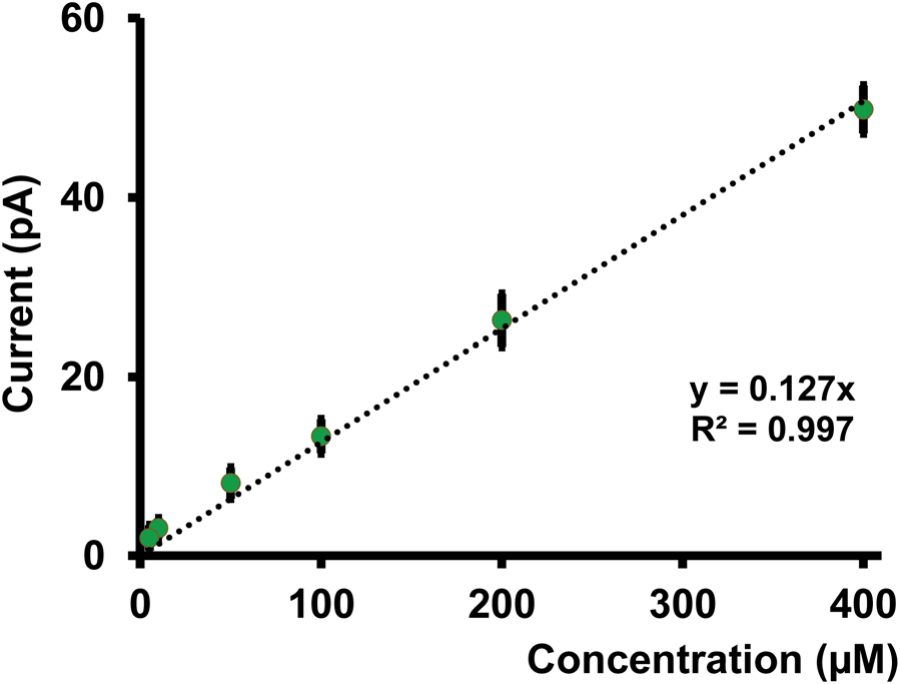
Calibration curve in ASW for Cd(ii) transfer. Each data point represents the average current ± standard error of the mean obtained for 4 nanopipets with at least 3 replicates for each pipet (minimum of 12 replicates in total) aqueous phase: *x* μM Cd(ii) in ASW buffer, where *x* = 5, 10, 50, 100, 200 and 400. Organic phase: 10 mM phen and 0.1 M TDDATFAB.

We performed calibration studies in KCl, tris buffer (Fig. S5 and S6 in ESI†), and ASW (Fig. 4) to find the sensitivity and LOD of our nanopipets. As seen in Fig. 4, the sensitivity in ASW was 0.127 pA μM^−1^ whereas in KCl, it was 0.162 pA μM^−1^ (Fig. S5 in ESI†). Although there is a notable change in the solution composition, the sensitivities are not drastically different; thus, the matrix effect from ASW on our sensor's performance is negligible. However, we noticed the sensitivity in tris buffer was much lower (0.085 pA μM^−1^) compared to the other two solutions; 1.5 times and 2 times lower compared to ASW and KCl, respectively. Furthermore, LOD in both KCl and ASW was 5 μM (0.56 ppm), whereas, in tris buffer, it was 10 μM (1.12 ppm). Moreover, a relatively high background current was obtained in tris buffer (Fig. S6 in ESI†). We ascribe the higher LOD and reduced sensitivity in tris to its solution composition that yields a high background current. Moreover, studies have reported that Cd(ii) makes strong complexes with tris.^23^ Thus, removing some free Cd(ii) ions in the solution subsequently decreases the measured current in our potential window. Based on these results, we performed most of our experiments in ASW, thus making our sensor the first nanopipet based electrochemical sensor that can detect Cd(ii) in complicated matrices.

Previously reported ITIES based studies were performed with macropipets or macro ITIES^12–14^ in a simple electrolyte solution composed of one salt. Furthermore, these studies are primarily focused on theoretical aspects compared to applications of these sensors to aid in solving real-world problems; thus, no calibration studies were performed, nor was LOD reported. Conversely, our primary goal was to determine whether this sensor could be utilized to detect Cd(ii) samples first in environmental samples and then in biological samples (future studies).

**Fig. 5:**
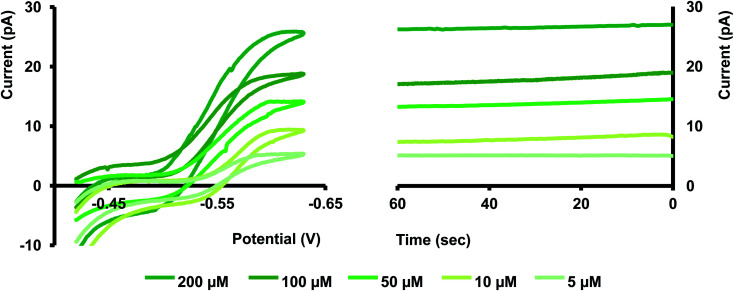
Left: representative CVs for different concentrations of Cd(ii) transfer in ASW. Right: current *vs.* time (*i*–*t*) traces for each concentration. Here, each *i*–*t* curve was obtained for 60 s by applying a constant potential obtained from the plateau region of the CVs.

We tested the stability of our sensor amperometrically by holding each nanopipet at the plateau-potential. Although the actual measurement requires only a few seconds, as seen in Fig. 5, the current was stable for over a minute. Maintaining a constant current at nano-ITIES is often challenging, mainly due to fouling that occurs from the adsorption of nanoscopic particles at the ultra-small interface. Our finding showcases the robustness of our sensor to overcome that obstacle, thus making it an excellent tool for real sample analysis. Moreover, our data is in good agreement with a similar study performed by Colombo *et al.*; hence, validating our sensor's stability.^21^

We also assessed the selectivity of our nanopipet towards Cd(ii) ions over some potential interfering ions; Co(ii), Fe(ii), Fe(iii), Ni(ii), Pb(ii), Ca(ii), Cu(ii), and Mg(ii) (Fig. S7 in ESI†). Here, we tested each individual ion (400 μM; upper limit of our linear range) in KCl using phen (10 mM). Although it is known that phen is not selective only for Cd(ii) ions,^24^ the rationale for this experiment was that *E*_1/2_ for each metal ion would be unique; therefore, it is still possible to distinguish Cd(ii) ions from other ions. Interestingly, only Cu(ii), Ca(ii), and Pb(ii) resulted in quasi-steady state CVs. However, all three metal ions showed a significantly lower current and high negative *E*_1/2_ compared to Cd(ii) (Table S1 in ESI†). Other metal ions didn't significantly respond within the potential limit we tested for Cd(ii). This is an exciting finding as our nanopipet can differentiate Cd(ii) with appreciable selectivity even using a non-selective ionophore.

### Cd(ii)–ligand complexes

**Fig. 6:**
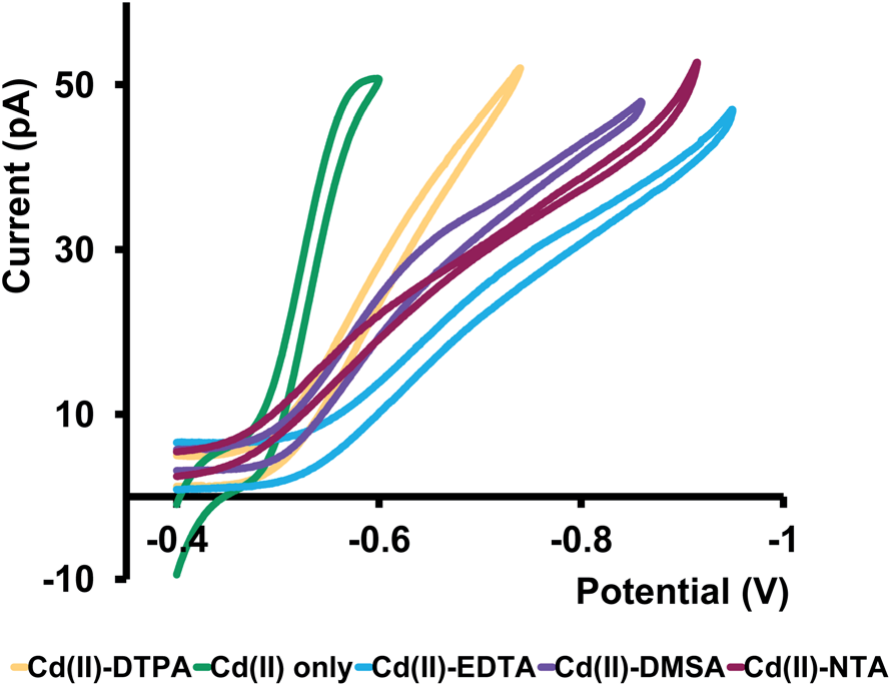
Representative CVs obtained for Cd(ii) ion transfer in the absence (green) and in the presence of EDTA (light-blue), DTPA (yellow), NTA (dark purple), and DMSA (light purple) in ASW at pH 7.0. Aqueous phase: 400 μM Cd(ii) and 400 μM ligand. Organic phase: 10 mM phen and 0.1 M TDDATFAB. All CV measurements were performed at 10 mV s^−1^.

Metals in environmental samples exist in complexed forms with naturally present ligands, thus lowering the concentration of free metal ions. However, free metal ions are readily accessible for chemical reactions, and therefore are more responsible for toxicity. Hence, it is vital to detect free metal ions in a system compared to bound metal ions. Before we tested our nanopipets with natural water samples, we performed a pilot study to verify our sensor's ability to detect free Cd(ii) ions in the presence of known ligands. We chose EDTA, DTPA, DMSA, and NTA as four model ligands. These were chosen based on their usage as Cd(ii) detoxifying agents in the medical field. These ligands have been tested for their efficacy by several research groups, primarily to administer them to remove ingested Cd(ii) from the body.^25–29^ We first mixed Cd(ii) and each ligand in 1 : 1 molar ratio in ASW and let the mixtures equilibrate for ∼24 h. Then we analyzed these samples with our nanopipets by running CV experiments. At pH 7, deprotonated forms^30–32^ of ligands exist as EDTA^3−^, DTPA^3−^, NTA^3−^ and DMSA^2−^; thus, we expected to observe prominent CVs corresponding to [Cd–EDTA]^−1^, [Cd–DTPA]^−1^, [Cd–NTA]^−1^ appearing with an increase in the negative current on the forward scan, and almost no CV for [Cd–DMSA]^0^ since the overall charge of the complex is zero at pH 7.0. However, as seen in Fig. 6, we observed quasi-steady-state CVs for all four Cd–ligand mixtures resembling positively charged, free Cd(ii) ions in ASW. We expanded our potential window beyond the limits shown in Fig. 6 to observe any [Cd–ligand] complexes; however, no such feature was observed for any of these complexes (Fig. S8 in ESI†).

Furthermore, the current at the quasi-steady state is lower, and CVs are less steep than the CVs collected for Cd(ii) in ASW. Interestingly, the *E*_1/2_ of all four samples shifted to higher negative potentials compared to that of Cd(ii). Therefore, we attribute these CVs to free Cd(ii) ions in equilibrium with Cd–ligand complexes. Lower current, less steepness, and higher *E*_1/2_ values are presumably due to the competition between the ligands and the applied potential. The presence of phen in the organic phase will favor the movement of free Cd(ii) ions to the organic phase, pulling more free Cd(ii) ions from the Cd–ligand complex. Moreover, because Cd(ii) ions are bound to ligands, the required energy for their ion transfer is higher than that in the absence of ligands; thus, *E*_1/2_ values are shifted more towards the high negative potentials. To the best of our knowledge, this is the first time reporting a Cd(ii)–ligand study with an ITIES-based nanopipet. This is an exciting finding as it showcases the power of our sensor to identify free Cd(ii) ions in the presence of strong complexing agents.

### Analysis of a real environmental sample

**Fig. 7:**
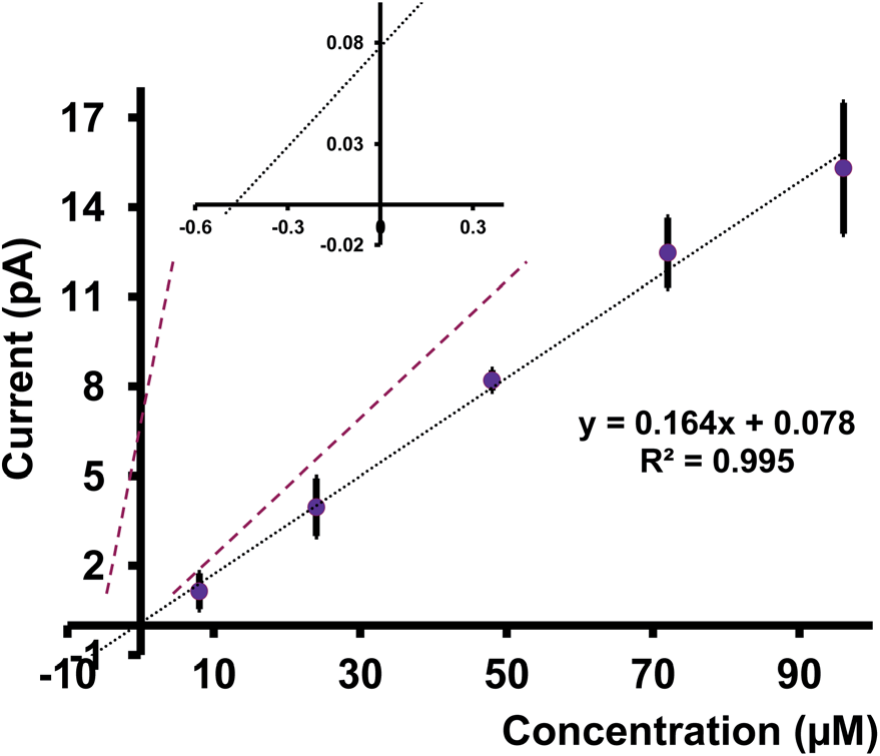
Plot between the concentration of Cd(ii) ions added and the steady-state current. Each data point represents the average current ± standard error of the mean obtained for 4 nanopipets with at least 3 replicates for each pipet (minimum of 12 replicates in total). Inset shows where the graph meets *X*-axis; thus, Cd(ii) concentration in the analyte sample.

We tested a water sample obtained from the Indian River Lagoon, Melbourne, FL, with our nanopipets. Here, we quantified the concentration of Cd(ii) ions in this water sample using the standard addition approach. We first let the river water sample settle for ∼48 hours and removed debris by filtration. A series of Cd(ii) concentrations were prepared by mixing a known volume of filtered river water sample with different concentrations of Cd(ii) in ASW (see Table S2 in ESI† for more details). The corresponding CVs were taken with at least 4 nanopipets, each with three runs. As depicted in Fig. 7, the average current for at least 12 replicates for each concentration was plotted against the concentration of Cd(ii). The extrapolation of the graph yielded the concentration of Cd(ii) in the analyte sample, and by adjusting the dilution factor, we found the concentration of Cd(ii) in the Indian River Water Lagoon to be 0.27 ppm. Interestingly, our finding agrees well with the literature. Trefry and Trocine reported Cd(ii) concentrations (0.62–0.26 ppm) at different locations in the lagoon.^33^ Furthermore, the authors used inductively coupled plasma mass spectrometry as their analysis tool, thus confirming our sensor's great potential for use as an environmental monitoring tool.

## Conclusions

In this paper, we described the use of nanopipet based electrochemical sensor to detect Cd(ii) ions in aqueous samples. Our electrode is a borosilicate glass electrode with an inner radius of 300 nm. It follows a hemispherical diffusion regime, owing to its nanoscale interface that allows fast measurements. Phen was used to facilitate the Cd(ii) transfer across the nano-interface. We performed ITIES based cyclic voltammetry and amperometry experiments with our nanosensor in various matrices, including simple electrolytes like KCl and complicated buffer solutions such as artificial seawater and artificial cerebellum fluid. We also tested the strength of our sensor against other standard ligands such as EDTA, NTA, DTPA, and DMSA. We found out that our electrode shows excellent stability and can withstand the complex matrices without fouling, an attractive feature of an exemplary sensor. We tested our sensor with Cd(ii) dissolved in a water sample collected from the Indian River Lagoon, Melbourne, FL; thus, we showcase our sensor's power as an environmental monitoring tool. To the best of our knowledge, this is the first time reporting a glass electrode with a nanometer scale for Cd(ii) detection in a natural environmental sample using ITIES. Our ultra-small electrode will enable us to study the kinetics of ion transfer across ITIES; thus, allowing us to modify the sensor to enhance the sensitivity and selectivity in our future studies.

## Conflicts of interest

There are no conflicts to declare.

## Supplementary Material

RA-012-D1RA07655H-s001
