# Supplementary material for: Electrochemical detection of Cd(ii) ions in complex matrices with nanopipets
Source: RSC Adv. 2022 Jan 5;12(2):1077–83. doi: 10.1039/d1ra07655h (PMC8978973; doi:10.1039/d1ra07655h)
Supplement: RA-012-D1RA07655H-s001 [file RA-012-D1RA07655H-s001.pdf]

Electronic Supplementary Material (ESI) for RSC Advances.
This journal is © The Royal Society of Chemistry 2021

Supporting Information

Electrochemical Detection of Cd(II) Ions in Complex Matrices with Nanopipets

Muzammil M. N. Ahmed [a], Faieza S. Bodowara [a], Wendy Zhou [a], Juliana F. Penteado [a], Jessica L. Smeltz [a], Pavithra Pathirathna*[a]

[a] Department of Biomedical & Chemical Engineering & Science, Florida Institute of Technology, 150 W. University Blvd, Melbourne, FL, 32901, USA.

*Email: ppathirathna@fit.edu

Contents:

1. SEM image of nanopipet tip
2. Cd(II) CV in tris buffer
3. Cd(II) CV in PBS
4. Cd(II) CVs in ASW
5. Calibration curves in KCl and tris
6. Selectivity test
7. Expansion of potential window
8. Standard addition method

SEM image of nanopipet tip - After pulling the nanopipets, we imaged the pipet tips with SEM (JEOL JSM-6380LV (JEOL Ltd, Tokyo, Japan) to measure the pipet tip diameter accurately. As seen in Figure S1, the average tip diameter for our nanopipets was ~600 nm. We used this information to calculate the diffusion coefficient of Cd(II) in different media.

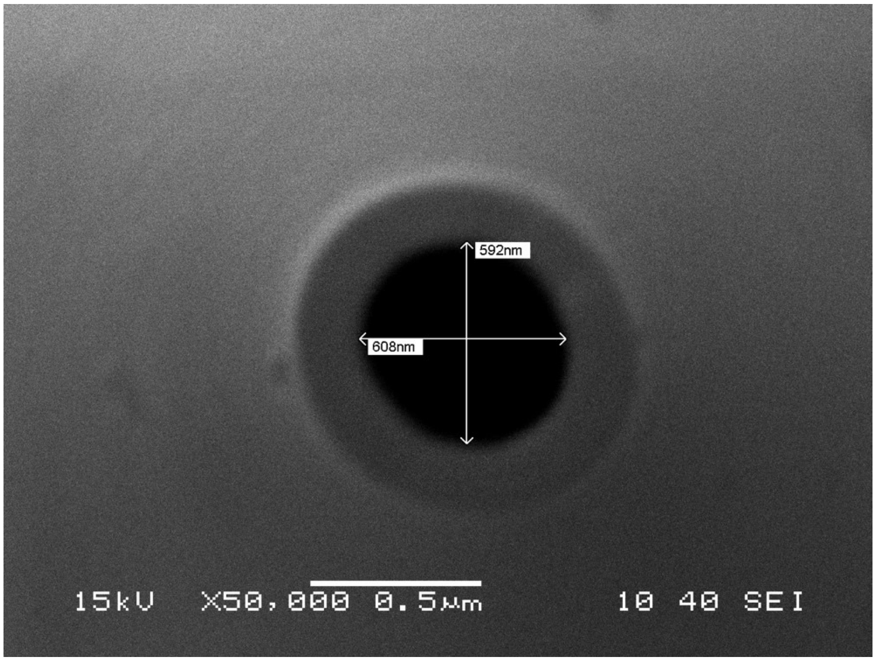

Figure S1: SEM image of nanopipette

Cd(II) CV in tris buffer - We tested tris buffer with our nanopipets as a potential matrix for our future studies. However, as shown in Figure S2, a relatively high background current was observed for the tris buffer. We ascribe this to the high concentrations of anions and cations present in the matrix. Conversely, the response for Cd(II) dissolved in tris buffer was comparatively low. This is presumably due to the complexation of Cd(II) ions with tris.

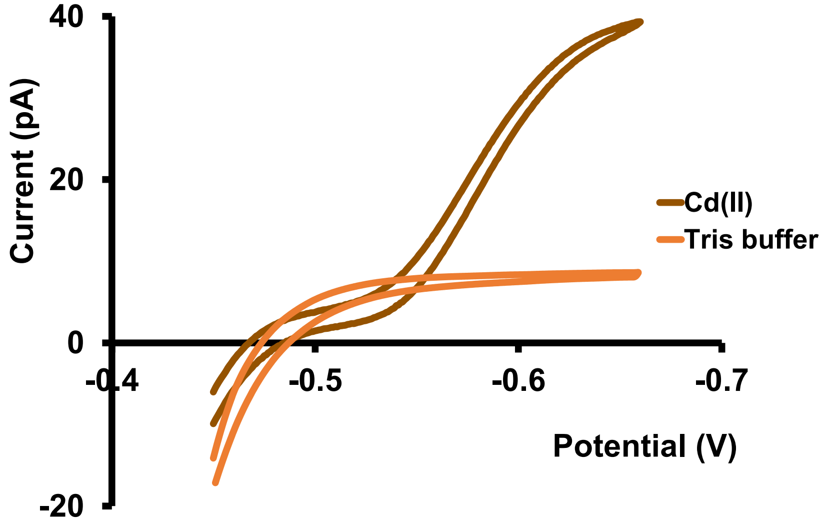

Figure S2: Brown trace is a representative CV of Cd(II) transfer between water and DCE at 10 mV s^{-1}. Aqueous phase: 400 µM Cd(II) in tris. Organic phase: 10 mM phen and 0.1 M TDDATFAB. Orange trace represents the background CV obtained with tris buffer only under the same experimental conditions.

Cd(II) CV in PBS - We also tested PBS as another potential matrix (Figure S3). However, the response of Cd(II) in PBS was very low compared to all other matrices we tested. This is most likely due to the formation of insoluble Cd-phosphate complexes.

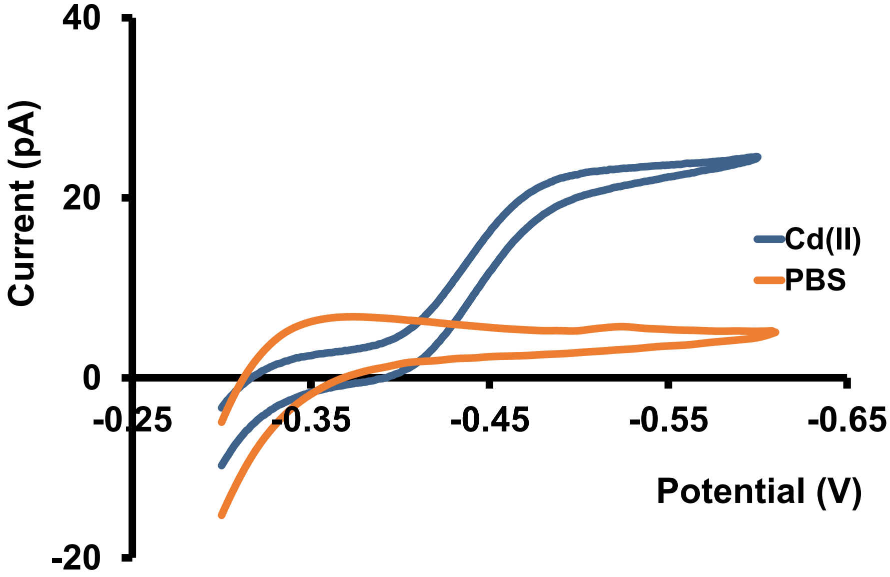

Figure S3: Dark blue trace is a representative CV of Cd(II) transfer between water and DCE at 10 mV s^{-1}. Aqueous phase: 400 µM Cd(II) in PBS. Organic phase: 10 mM phen and 0.1 M TDDATFAB. Orange trace represents the background CV obtained with PBS only under the same experimental conditions.

Cd(II) CVs in ASW - We repeated every experiment with at least four nanopipets to check the reproducibility of our sensors (Figure S4). Each nanopipet had different background currents (data not shown here) and was subtracted from each CV. Moreover, we attribute the slight potential shift in $E_{1/2}$ values of different pipetes to the small fluctuations in the potential of our lab-build reference electrode.

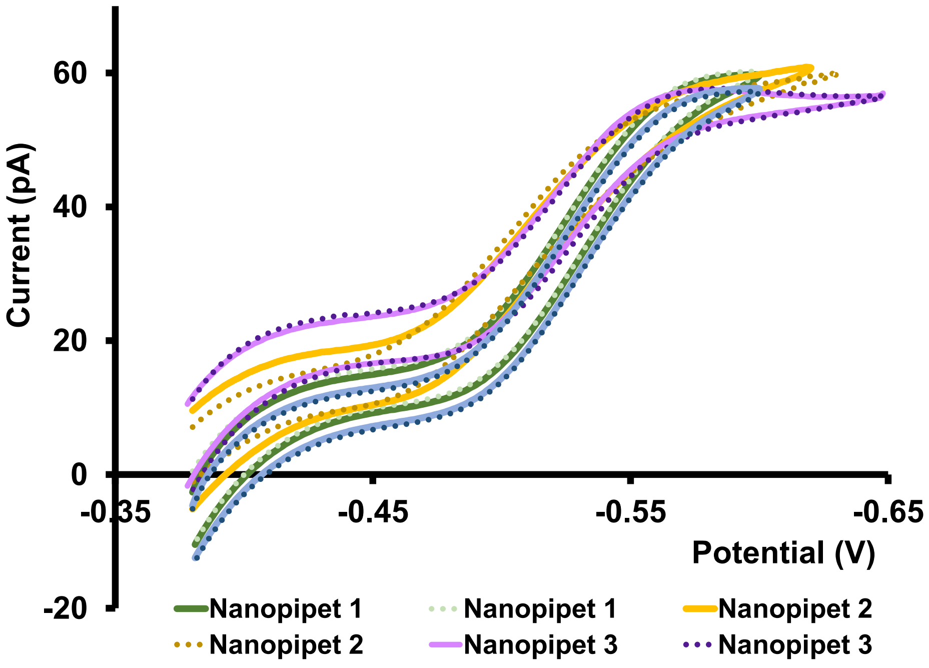

Figure S4: Replicate CVs of Cd(II) transfer in ASW. Aqueous phase: 400 µM Cd(II) in ASW buffer, Organic phase: 10 mM phen and 0.1 M TDDATFAB.

Calibration curve in KCl and Tris - We constructed calibration curves in KCl and tris buffer in addition to the one in ASW. As seen in Figure S5, the calibration in KCl is more or less similar to that of ASW. However, the curve in tris buffer (Figure S6) has a large intercept and small slope, indicating, relatively, a significant effect from the matrix.

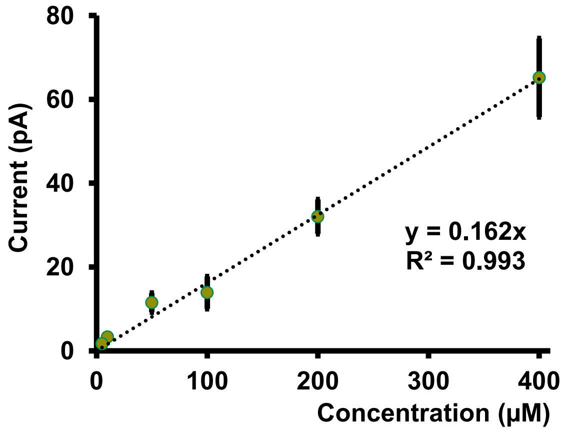

Figure S5: Calibration curve in KCl for Cd(II) transfer. Each data point represents the average current ± standard error of the mean obtained for 4 nanopipets with at least 3 replicates for each pipet (minimum of 12 replicates in total). Aqueous phase: x µM Cd(II) in KCl, where x = 5, 10, 50, 100, 200 and 400. Organic phase: 10 mM phen and 0.1 M TDDATFAB**.**

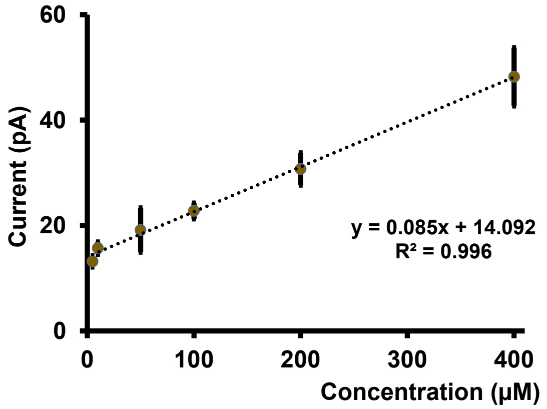

Figure S6: Calibration curve in tris buffer for Cd(II) transfer. Each data point represents the average current ± standard error of the mean obtained for 4 nanopipets with at least 3 replicates

for each pipet (minimum of 12 replicates in total). Aqueous phase: x µM Cd(II) in tris buffer, where x = 5, 10, 50, 100, 200 and 400. Organic phase: 10 mM phen and 0.1 M TDDATFAB**.**

Selectivity test – We performed a selectivity test with a series of other potentially interfering metal ions; Co(II), Fe(II), Fe(III), Ni(II), Pb(II), Ca(II), Cu(II), and Mg(II). A seen in Figure S7, and Table S1, Cu(II), Ca(II), and Pb(II) resulted in quasi-steady state CVs with different $E_{1/2}$ values and lower current compared to Cd(II). In contrast, others didn't show any significant response.

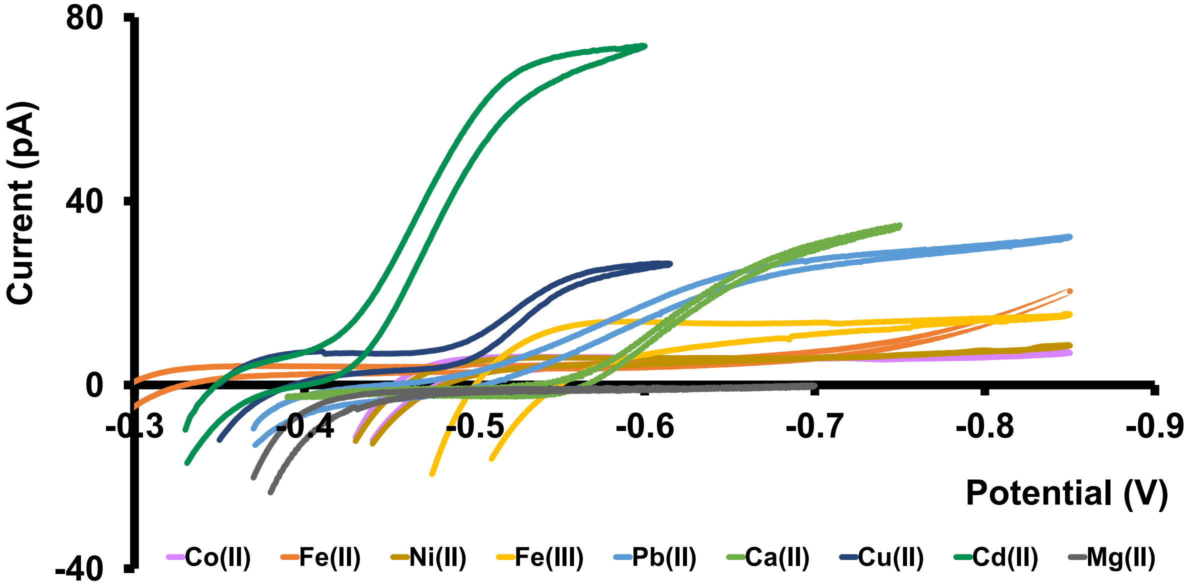

Figure S7: Representative CVs for the transfer of interfering metal ions between water and DCE at 10 mV s^{-1}. Aqueous phase: 400 µM M(II/III) in KCl. Organic phase: 10 mM phen and 0.1 M TDDATFAB.

Table S1: $E_{1/2}$ values for each metal ion

Metal ion	Cd(II)	Cu(II)	Pb(II)	Ca(II)	Fe(III)	Fe(II)	Ni(II)	Mg(II)	Co(II)
$E_{1/2}$ (V)	-0.46	-0.53	-0.58	-0.62	No response	No response	No response	No response	No response

Expansion of the potential window - The net charge of all [Cd-ligand] complexes, except [Cd-DMSA]^0, was -1 under our experimental conditions; thus, we expected to observe CVs resembling the transfer of negative ions from the aqueous phase to the organic phase. Interestingly, the CVs we obtained were similar to that of Cd(II) ions. To further validate our observation, we expanded the potential window to confirm that no additional quasi-steady-state CVs appear with a negative current. As seen in Figure S8, currents at the two ends are significantly larger (nA range) than what we would expect for our Cd-ligand complexes. These large currents originate from high concentrations of other ions in the matrix; thus, it confirms that we can only detect the quasi-steady-CVs of free Cd(II) ions even in the presence of strong complexing ligands.

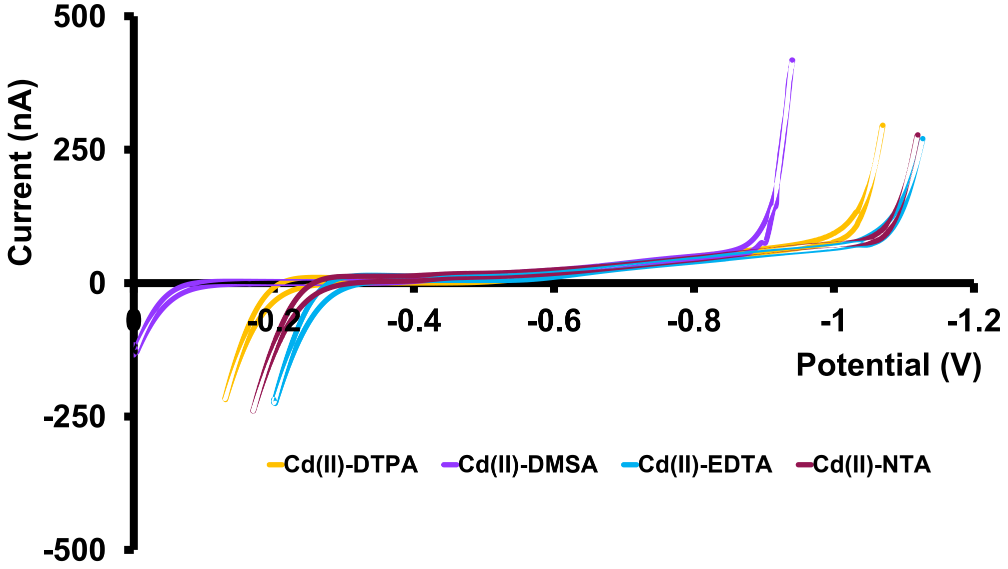

Figure S8: CVs of [Cd-ligand]^n transfer and between water and DCE at 10 mV s^{-1} (where n=-1 or 0). Aqueous phase: 400 µM Cd(II) in ASW. Organic phase: 10 mM phen and 0.1 M TDDATFAB.

Standard addition method - We quantified the concentration of Cd(II) ions in the Indian River Lagoon using the standard addition method. Table S2 shows the experimental parameters we used to perform this experiment.

Table S2: Experimental parameters used for standard addition method

Sample no.	Volume of 400 µM Cd(II) solution (mL)	Volume of river water sample (mL)	Total volume (mL)	Final conc. of Cd(II) (µM)
1.	1	10	50	8.5
2.	3	10	50	24.5
3.	6	10	50	48.5
4.	9	10	50	72.5
5.	12	10	50	96.5
